# Individual Perception of Telehealth: Validation of a German Translation of the Telemedicine Perception Questionnaire and a Derived Short Version

**DOI:** 10.3390/ijerph19020902

**Published:** 2022-01-14

**Authors:** Patrick Altmann, Dominik Ivkic, Markus Ponleitner, Fritz Leutmezer, Ulrike Willinger, Michaela Schmoeger, Thomas Berger, Gabriel Bsteh, Henriette Löffler-Stastka

**Affiliations:** 1 Department of Neurology, Medical University of Vienna, Waehringer Guertel 18-20, 1090 Vienna, Austria; patrick.altmann@meduniwien.ac.at (P.A.); dominik.ivkic@outlook.com (D.I.); markus.ponleitner@meduniwien.ac.at (M.P.); fritz.leutmezer@meduniwien.ac.at (F.L.); ulrike.willinger@meduniwien.ac.at (U.W.); michaela.schmoeger@meduniwien.ac.at (M.S.); thomas.berger@meduniwien.ac.at (T.B.); 2 Department of Psychoanalysis and Psychotherapy, Medical University of Vienna, 1090 Vienna, Austria; henriette.loeffler-stastka@meduniwien.ac.at

**Keywords:** telemedicine, telehealth, satisfaction, perception, questionnaire

## Abstract

Telehealth is a growing domain with particular relevance for remote patient monitoring. With respect to the biopsychosocial model of health, it is important to evaluate perception and satisfaction with new methods in telehealth as part of an integrative approach. The Telemedicine Perception Questionnaire (TMPQ) is a 17-item questionnaire measuring patients’ perception of and satisfaction with telecare. We translated this survey into German and determined its validity and reliability in 32 adolescents and adults. Furthermore, we derived a short version of the TMPQ, named Patient and Physician Satisfaction with Monitoring (PPSM), which is a 5-item questionnaire that can be administered to both patients and physicians. Validity and reliability were tested in 32 patients and 32 physicians. Crohnbach’s α for the translated TMPQ was 0.76, and the German version yielded high validity (intraclass correlation coefficient (ICC) 0.995). We tested the PPSM in both patients and physicians and found acceptable values for Crohnbach’s α (0.72 and 0.78) with excellent validity (ICC 0.965). We therefore concluded from this small study that both German versions of the TMPQ and PPSM can be used to investigate the acceptance of telehealth applications.

## 1. Introduction

Telehealth is a domain of information and communication technology in medicine and constitutes an exchange of health information without a physical co-presence of the patient and physician [1]. The provision of remote healthcare is the hallmark of telehealth. The field of telehealth surrounds elements of increasing access to healthcare, ensuring medical care from home and developing concepts for remote care in acute as well as chronic conditions. Thus, components of telehealth potentially complement the biopsychosocial model of health, and may foster an integrative approach in patient care [2,3]. Particularly in chronic conditions, remote patient monitoring combines all of these three aspects, as the patient’s health status can be documented and monitored off-site. In this context, it is important to understand and investigate the perception and experience of patients who access remote health services [4,5]. Furthermore, in the face of the current global pandemic, contemporary telehealth concepts are particularly pertinent. Chronically ill patients, above all, need tele services to keep in contact with their treating physicians. Above all, telehealth applications may become more and more relevant when studying patient outcomes, particularly in people with chronic diseases.

The Telemedicine Perception Questionnaire (TMPQ) is a 17-item survey relevant to telehealth, as it measures the patients’ acceptability of telemonitoring in terms of their perception of the risks and benefits [6]. Its use allowed us to rate the design and implementation of telehealth systems, and underline its value in assessing new strategies in telemedicine. The TMPQ is currently the only tool that is licensed and, thus, free to use for academic purposes [7]. It has been validated and is already used in a study for web chat triage [8].

The aim of the present study was to validate, in a small cohort, a German translation of the TMPQ and evaluate a new short 5-item survey, addressing satisfaction with remote monitoring. This short survey is the Patient and Physician Satisfaction with Monitoring (PPSM) questionnaire, and it can be administered to both patients and physicians.

## 2. Materials and Methods

The validation process of both the TMPQ (translation and validation) and the PPSM (validation) followed specific guidelines for developing, translating, and validating a questionnaire [9]. Validity was investigated based on content validity and construct validity in the sense of criterion validity. Tests for reliability included internal consistency and test–retest reliability.

The TMPQ was not available in German, and underwent forward and backward translation. The use of the survey was approved by its inventor [6]. The original English version was translated to German by two native German health scientists, discussed by our staff (three neurologists, two clinical psychologists, one psychiatrist) and modified accordingly. The backward translation from German to English was performed by an independent native English translator, and discussed by the staff mentioned above. The German translated version can be made available upon request. As for content validity, the expert panel decided the questions were clear and easy, covered appropriate problem areas, and confirmed usefulness for investigators. Construct validity in the sense of criterion validity was established by comparing the overall means and variances of answers given on the study of the original survey with the translated version. Internal consistency is given as the overall Crohnbach’s α in the translated version along with Crohnbach’s α for single items left out. All participants in this validation study filled out the survey at baseline and after two weeks. Test–retest reliability was determined by calculating the intraclass correlation coefficients (ICC) between answers given at both points in time.

The PPSM questionnaire was derived from the TMPQ and adapted so that the questions can apply to both health care professionals (e.g., doctors or nurses) and participants (e.g., patients). It consists of five questions concerning the following dimensions: (i) perception of health; (ii) privacy; (iii) time effectiveness; (iv) perceived usefulness; and (v) general satisfaction. The PPSM survey was validated in German, a suggestion for an English translated version is shown in Table 1. An expert panel was assembled consisting of three neurologists, two clinical psychologists, and one psychiatrist. They decided on the dimensions named above and reviewed and revised the questions resulting in six questions to be validated in pilot testing. For the final five questions, the expert panel decided the questions were clear and easy, covered appropriate problem areas, and confirmed usefulness for investigators. We decided to use the same 5-point-Likert scale as in the original TMPQ (strongly agree (5 points), agree (4 points), no opinion (3 points), disagree (2 points), strongly disagree (1 point)). Criterion validity was established by comparing the overall means and variances of answers given on the original TMPQ validation study and the new PPSM. All participants in this validation study filled out the survey at baseline and after two weeks. Test–retest reliability was determined by calculating the ICC between answers given at both points in time.

The TMPQ and PPSM surveys were both administered to 32 patients at the multiple sclerosis outpatient clinic of the Department of Neurology at the Medical University of Vienna, Austria, between 1 August 2020 and 1 April 2021. These patients experienced remote patient monitoring through the use of a smartphone application that tracked patient-reported outcomes. The PPSM was also administered to 32 physicians of the Department of Neurology at the Medical University of Vienna, Austria, between 18 January and 19 February 2021. The physicians that were surveyed shared their experience based on performing teleconsultations over the phone or via email.

## 3. Results

### 3.1. TMPQ

A total of 32 participants took part in this study, 56% of the participants were female, and the median age was 32 years (interquartile range [IQR]: 27–39). Regarding reliability, the German version of the TMPQ displayed an overall Cronbach’s α of 0.76, versus 0.8 in the original study. The item-per-item analysis did not suggest the deletion of any item (Table 2). Test–retest reliability was high for all items. Construct validity analysis showed a strong correlation (ICC 0.995) between the German version (mean score: 3.75, standard deviation [SD]: 0.44) and the English version (mean score: 3.78, SD: 0.46; *p* = 0.453 (paired *t*-test)) derived from published data [6].

### 3.2. PPSM: Patients

For the short PPSM survey applied to the same 32 patients as the TMPQ, Crohnbach’s α was found to be 0.72. The item-per-item analysis did not suggest the deletion of any item (Table 3). Test–retest reliability was excellent for all items. Construct validity analysis using the TMPQ as the gold standard showed a strong correlation (ICC 0.928, *p* = 0.002) between the TMPQ (mean score: 3.78, SD: 0.46) and PPSM (mean score 3.66, SD: 0.29; *p* = 0.338 (paired *t*-test)).

### 3.3. PPSM: Physicians

In total, 32 physicians took part in this validation, out of which 53% were male and the median age was 33 years (IQR: 28–40). Overall, Crohnbach’s α of the PPSM in physicians was 0.78. Deleting item number 2 would have resulted in a greater internal consistency, yet we decided to leave it in, as it carries an important piece of information (Table 4). Test–retest reliability was good to excellent for all items. Construct validity compared to the TMPQ as the gold standard was excellent (ICC 0.965, *p* = 0.002).

## 4. Discussion

This study aimed to validate a German version of the TMPQ and introduce a short version of it named PPSM, that addresses both the patient’s and physician’s perception of remote monitoring as part of telehealth. The German version of the TMPQ yielded a high level of internal consistency and test–retest reliability, as well as excellent construct validity compared with the original version. Crohnbach’s α was 0.76. In addition, we developed a short survey for reception and satisfaction with monitoring named PPSM. The PPSM questionnaire can be administered to both patients and treating physicians. We found sufficient values for internal consistency (Crohnbach’s α in patients was 0.72 and 0.78 in physicians) as well as excellent test–retest reliability and construct validity.

This study concerning the validation of the TMPQ translation has some limitations. First, the sample size at *n* = 32 was small. Upon conception of this study, there were no comparable investigations using or validating a German survey for the individual perception of telehealth. This is why we mirrored the sample size from the original validation study [6]. In addition, we referred to an approximation formula that suggested the required sample size for calculating a meaningful Crohnbach’s α at *n* = 18 [10]. That being said, the conclusions from this study must be interpreted within the border of this limitation. Second, our sample differed from the original English validation cohort, a common issue with translating questionnaires. The population in which the validity was tested for the original study consisted of residents of an assisted-living facility for the elderly. In our study, we referred to patients visiting our neurology outpatient clinic. However, we think that our participants were representative and relevant as they experienced telemonitoring through the use of a smartphone application serving as a tool for remote monitoring. The same goes for the sample of patients in which the PPSM was tested. The physicians questioned for the validation of the new PPSM survey were filling out the questionnaires based on their experience concerning the exchange of health information with their patients over the phone or via email.

The PPSM is a new questionnaire, and studies to test its usability and acceptability in a broader population could further demonstrate its applicability, e.g., testing this survey in distinct age groups and diagnoses for patients and different medical specialties for physicians. Even so, the recruited cohorts for the PPSM validation, both patients and physicians, are representative with respect to direct involvement in telemonitoring for which the questionnaire was developed. It is important to stress the relevance of convenient surveys investigating the perception of telehealth applications. In the future, telehealth may aid in gathering real-world evidence, particularly in the care for patients with chronic conditions.

In summary, the translation of the 17-item TMPQ into German was successful. Framing this validation in the light of small sample size, we can encourage the use of the TMPQ in German studies that investigate patients’ perception and satisfaction with telecare. The development of the German 5-item PPSM questionnaire yielded sufficient results for reliability and validity. Its use is particularly interesting for telehealth applications that require grading from both patients and physicians. In the future, these questionnaires may help integrate information from remote patient monitoring into the biopsychosocial model of health.

## Figures and Tables

**Table 1:** The PPSM survey.

1	This method gives the treating physician a good understanding of the patient’s health status.
2	This method does not violate privacy of the patient’s medical information.
3	This method is a good addition to regular care.
4	This method saves time.
5	I would use this method in the future.

**Table 2:** Internal Consistency and test–retest reliability for the translated TMPQ.

(a) Internal Consistency		(b) Test-Retest Reliability for the Translated TMPQ			
Internal Consistency		Test–Retest Reliability			Item Total Correlation
Item No.	α If Deleted	ICC	95%CI	*p*-Value	
1	0.74	0.75	0.44–0.88	<0.001	0.44
2	0.77	0.76	0.43–0.84	<0.001	0.06
3	0.75	0.70	0.38–0.85	<0.001	0.38
4	0.75	0.71	0.42–0.86	<0.001	0.31
5	0.75	0.60	0.19–0.80	0.008	0.27
6	0.75	0.62	0.25–0.81	0.003	0.33
7	0.72	0.60	0.15–0.80	0.009	0.56
8	0.75	0.84	0.67–0.92	<0.001	0.31
9	0.80	0.60	0.18–0.80	0.007	0.31
10	0.72	0.87	0.73–0.93	<0.001	0.66
11	0.74	0.86	0.71–0.92	<0.001	0.43
12	0.72	0.79	0.56–0.90	<0.001	0.56
13	0.76	0.80	0.58–0.90	<0.001	0.16
14	0.76	0.86	0.70–0.91	<0.001	0.24
15	0.73	0.71	0.42–0.86	<0.001	0.51
16	0.71	0.81	0.62–0.90	<0.001	0.67
17	0.74	0.60	0.24–0.79	0.006	0.41

‘α if deleted’ refers to Crohnbach’s α with items left out. ICC: intraclass correlation coefficient. 95%CI: 95% confidence interval.

**Table 3:** Internal consistency and test–retest reliability for the PPSM survey in patients.

(a) Internal consistency		(b) Test-Retest Reliability for the PPSM Survey in Patients		
Internal Consistency		Test–Retest Reliability		
Item No.	α If Deleted	ICC	95%CI	*p*-Value
1	0.669	0.92	0.83–0.96	<0.001
2	0.662	0.91	0.81–0.96	<0.001
3	0.694	0.95	0.91–0.98	<0.001
4	0.658	0.95	0.89–0.97	<0.001
5	0.587	0.85	0.70–0.93	<0.001

‘α if deleted’ refers to Crohnbach’s α with items left out. ICC: intraclass correlation coefficient. 95%CI: 95% confidence interval.

**Table 4:** Internal consistency and test–retest reliability for the PPSM survey in physicians.

(a) Internal Consistency		(b) Test-Retest Reliability for the PPSM Survey in Physicians		
Internal Consistency		Test–Retest Reliability		
Item No.	α If Deleted	ICC	95%CI	*p*-Value
1	0.70	0.92	0.81–0.96	<0.001
2	0.83	0.94	0.87–0.97	<0.001
3	0.70	0.88	0.75–0.94	<0.001
4	0.78	0.92	0.84–0.96	<0.001
5	0.69	0.87	0.73–0.94	<0.001

‘α if deleted’ refers to Crohnbach’s α with items left out. ICC: intraclass correlation coefficient. 95%CI: 95% confidence interval.

## Data Availability

Datasets can be requested upon reasonable request.
